# Balancing the personal, local, institutional, and global: multiple case study and multidimensional scaling analysis of African experiences in addressing complexity and political economy in health research capacity strengthening

**DOI:** 10.1186/1478-4505-13-5

**Published:** 2015-01-17

**Authors:** Alastair Ager, Christina Zarowsky

**Affiliations:** Mailman School of Public Health, Columbia University, 116th St & Broadway, New York, NY 10027 USA; School of Public Health, University of Western Cape, Robert Sobukwe Road, Bellville, Cape Town, 7535 South Africa; Centre de Recherche du Centre Hospitalier de l’Université de Montréal (CR-CHUM), 850, rue St-Denis, Montreal, (Québec) H2X 0A9 Canada

**Keywords:** Capacity strengthening, Complexity, Global health research, Globalization, Institutional capacity, North-South-South partnerships, Political economy of health research, Mentorship

## Abstract

**Background:**

Strengthening health research capacity in low- and middle-income countries remains a major policy goal. The Health Research Capacity Strengthening (HRCS) Global Learning (HGL) program of work documented experiences of HRCS across sub-Saharan Africa.

**Methods:**

We reviewed findings from HGL case studies and reflective papers regarding the dynamics of HRCS. Analysis was structured with respect to common challenges in such work, identified through a multi-dimensional scaling analysis of responses from 37 participants at the concluding symposium of the program of work.

**Results:**

Symposium participants identified 10 distinct clusters of challenges: engaging researchers, policymakers, and donors; securing trust and cooperation; finding common interest; securing long-term funding; establishing sustainable models of capacity strengthening; ensuring Southern ownership; accommodating local health system priorities and constraints; addressing disincentives for academic engagement; establishing and retaining research teams; and sustaining mentorship and institutional support. Analysis links these challenges to three key and potentially competing drivers of the political economy of health research: an enduring model of independent researchers and research leaders, the globalization of knowledge and the linked mobility of (elite) individuals, and institutionalization of research within universities and research centres and, increasingly, national research and development agendas.

**Conclusions:**

We identify tensions between efforts to embrace the global ‘Community of Science’ and the promotion and protection of national and institutional agendas in an unequal global health research environment. A nuanced understanding of the dynamics and implications of the uneven global health research landscape is required, along with a willingness to explore pragmatic models that seek to balance these competing drivers.

## Background

Health research capacity strengthening (HRCS) in the global South has become a major development objective, backed by arguments for the importance of national health research capacity to support locally-relevant, evidence-based health policy and practice [1–5]. This paper draws upon a program of research and reflection on the dynamics of HRCS which engaged with initiatives across Southern, Eastern, and West Africa. The HRCS Global Learning (HGL) program of work compiled inventories of initiatives to support research capacity strengthening at individual, institutional, and systems levels across the continent [6] and mapped institutions engaged in health research training [7–9]. It also commissioned critical reviews of institutional arrangements for HRCS as well as evaluative reflections of experiences in research capacity strengthening in public health, including cross-cutting themes such as gender and power [10–17], analyses focusing on national and regional health research systems development [18, 19], and reflections by African health research leaders on key principles for supporting health research capacity development [20]. It was informed by symposia, workshops, and other activities supported through a linked International Development Research Centre (IDRC)-funded project, *Strengthening African Research for Responsive Health Policies and Systems* undertaken by the School of Public Health and Centre for Research in HIV and AIDS of the University of Western Cape (UWC), South Africa. This project included an international symposium on *Public Health in the Age of HIV* [21] and a research project and workshop on *Situating research in public health training and practice: current debates and emerging good practice* [17], with subsequent engagement in the development of the Association of Schools of Public Health in Africa. Our analysis also reflects the experience of ongoing UWC participation in other collaborative capacity strengthening initiatives funded by a range of funders, including the European Union (CHEPSAA), WHO, the Bill and Melinda Gates Foundation [22], the Commonwealth Foundation, Atlantic Philanthropies, IDRC, VLIR-UOS, and the President’s Emergency Plan for AIDS Relief through the US Centers for Disease Control and Prevention (PEPFAR/CDC). The individual reviews and analyses were collated into a series of 22 briefs, *Learning about Research Capacity Strengthening* [23].

Drawing on the individual and shared experience of the project leaders and co-authors – with their dual identities as academics in African and North American universities and as funders working with Canadian and UK agencies committed to research capacity strengthening – this paper reports on a thematic analysis of the multiple HGL outputs, driven by the questions: What common challenges are those engaged in HRCS confronting? What are the key drivers behind these challenges?

### Methodology

To assist in the structuring of this analysis, we utilized a consultation at the end of the HRCS Global program. The results of this consultation were triangulated against our interpretive thematic analysis across the full set of HGL outputs. This paper reports on the synthesized analysis and interpretation using the consultation results as a structuring heuristic. The symposium *Learning About Research Capacity Strengthening: Reflections on Challenges, Strategies and Culture* was held at the Global Forum for Health Research meeting in Cape Town in April 2012. Over 40 invited stakeholders – comprising participants in and funders and evaluators of HRCS programs – reflected upon common challenges and potential strategies in such work. Explicitly, the symposium discussion sought to deepen understanding of, and constructively engage with, the institutional, politico-cultural, and interpersonal dynamics that are experienced at all levels of capacity strengthening by people working in the field but which are seldom captured in proposals, work-plans, and output-based evaluations of capacity strengthening efforts.

After a period when participants were facilitated – through active group work – to recurrently reflect on recent experiences of HRCS, participants at the symposium were asked to suggest the three major challenges that faced those seeking to facilitate such work. Thirty-seven participants wrote brief descriptors of challenges on large ‘post-it’ notes. Each descriptor was considered a discrete item for analysis, collated after the meeting in the fashion described in more detail by Ager at al. [24].

The total of 110 items elicited were sorted by four independent raters (all of whom had been present at the meeting). Raters independently sorted items into piles on the basis of common themes, with no restriction on numbers of themes identified. For each rater a 110 × 110 concordance matrix was generated which signaled for all item pairs whether that rater had grouped them together (1) or apart (0). Concordance matrices for the four raters were then consolidated into a single 110 × 110 agreement matrix, which for each item pairing noted the proportion of raters that had grouped those two items together (providing a ratio between 0.0 – never, and 1.0 – always). This agreement matrix was then analyzed using multi-dimensional scaling (MDS) [25, 26]. MDS is an exploratory technique used to derive structures that represent relationships between items in a visual manner [27].

Analysis generated a two-dimensional map where proximity of items represented the probability that they were grouped together by raters (i.e., two items grouped together by all raters are positioned close together; two items never grouped together by any rater are positioned far apart; two items grouped together by some raters and not others are assigned intermediate proximity). The MDS map was reviewed to identify discrete clustering of items using indices of item density and proximity. The items comprising each of these clusters were then independently reviewed by three researchers who produced preliminary thematic labels – which were then consolidated into consensus themes – representing the content of that cluster.

## Results

**Figure 1:**
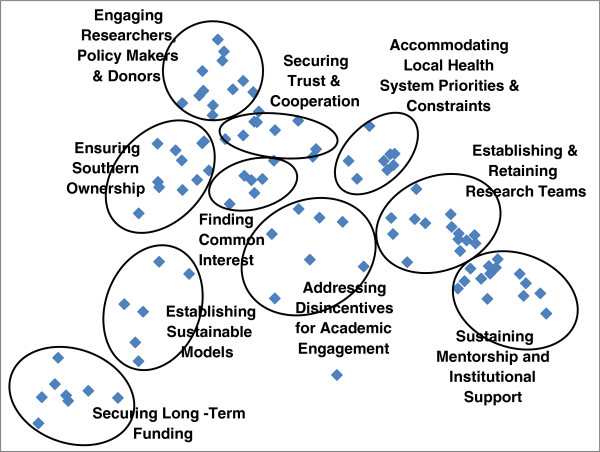
**Map of emerging clusters with assigned thematic labels.**

Figure 1 shows the clustering of items and the consensus labels for each thematic cluster. This two-dimensional map accounts for 80.7% of total variance in item grouping, and was thus deemed to represent a valid basis for interpretation of the overall patterning of items. The map identifies 10 distinct, though clearly related, challenges. Given that the proximity of clusters reflects the likelihood of items within them being grouped together, their positioning clearly suggests a basis for their interpretation. However, there are a number of ways of ‘reading’ the sequence of clusters, and pathways linking them should be considered multidirectional. Thus, while the clusters are considered in turn below in a broadly clockwise direction, this should not be taken to suggest a dominant sequence of causality.

### Securing long-term funding

A number of participants identified the long-term funding required for meaningful capacity strengthening as a major challenge. While for some it was the lack of availability of funds *per se* that presented difficulty, others noted it was the mismatch between the availability of short-term funding for specific research initiatives and the requirements for longer-term investment in capacity that was the principle source of difficulties. Many of the HGL case studies had noted this tension, including the review of HRCS in West Africa [15]. In another [13], Luna and Ager had analyzed the tensions of seeking to build long-term partnership for a North-South collaborative doctoral training program on the basis of a specific short-term funding source.

### Establishing sustainable models

Closely linked to the concerns of long-term funding was the establishment of sustainable models of health research capacity development. Sources of financing remained a central concern here (particularly addressing the lack of host government investment), but so too were time scales in expectations of research activity. Some participants noted the lack of interest in investing in ‘novice’ researchers, for example, while others observed that researchers were principally drawn by incentives ‘to consultancy not research’ (a theme elaborated later).

### Ensuring Southern ownership

The adjacent cluster of issues extended this concern to broader issues of Southern ownership. As one participant observed a key question is “*how to support Southern-led priorities when much of the funding focus is Northern/funder driven?*” Other contributory issues to this challenge were seen as weak South-South linkages (including lack of connection from Anglophone to Francophone and Lusophone contexts) and the loss of skilled researchers from the South (what one participant referred to as “*the South-North research capacity strengthening initiative*”).

### Engaging researchers, policymakers, and funders

Many responses reflected on the different interests of researchers, policymakers, and funders, and the difficulties in bridging between these agendas. Without honest exchange, and an acknowledgement of the differential power at work in seeking to resolve tensions in perspective, the notion of ‘equitable partnership’ was seen as illusory. The lack of a clear national research strategy was seen as an additional potential contribution to difficulties, with others pointing to the frequent lack of clear policy demand from policymakers in the health sector as a related constraint.

### Securing trust and cooperation

This cluster reflected statements developing this analysis of challenges in engagement with multiple stakeholders, with greater emphasis on issues of commitment, understanding, and trust. “*Managing expectations and maintaining trust through operational friction*” had emerged as one of the major themes of the case study of establishment of a collaborative, inter-institutional doctoral program [13], p. 2. Their analysis indicated that inter-individual trust was pivotal in negotiating periods where formal inter-organizational relationships faltered, with one respondent suggesting “*Institutions won’t trust each other; it is individuals that have to trust in each other*” [13], p. 29. Symposium participants echoed this theme with suggestions that contestations over understandings of appropriate approaches and associated ‘power battles’ regularly needed to be addressed, with another summarizing the core challenge as: “*getting various stakeholders involved in health research to trust each other and work together*”.

### Finding common interest

‘Finding common interest’ reflected a cluster of issues positioned at the fulcrum of the preceding three clusters and the one immediately following, and thus suggested as closely linked to these other concerns. The common theme was in relation to what one participant described as “*the value proposition for health research capacity strengthening that will persuade* [stakeholders] *to invest*”. Comments noted the lack of incentives for many stakeholders to engage in building capacity together, including “*monitoring and evaluation work being undervalued in academe*” and “*competition between* [both] *researchers and institutions*”, different and often mutually incomprehensible conceptual frameworks and terminologies across disciplines and the difficulties of integrating trainees whose formative experience was with ‘Northern’ institutions into local institutional and national capacity development strategies.

### Addressing disincentives for academic engagement

This cluster developed the analysis of disincentives specifically from the perspective of academics. Some were clear extensions of issues grouped in the preceding cluster such as ‘*the lack of recognition for knowledge transfer activities within academic career development*’. Others raised issues of the ‘*per diem culture’* that incentivized workshop attendance and, more generally, the lack of attractiveness of research as a career in many Southern contexts.

### Accommodating local health system priorities and constraints

This domain focused less on issues within academia and rather more on issues in relating research to the ‘real world’ environment of health systems in low-income settings. This included major constraints on capacity at district and provincial levels that undermined realistic expectations of local commissioning of research, and related difficulties of identifying ‘gaps’ in knowledge relevant to local implementation that could plausibly be filled by research. Acknowledging the broader drivers on the research foci of researchers, it was acknowledged that even if successfully articulated there may be low adherence to local health priorities. Whether at the national, district, or other levels, researchers’ professional advancement incentives (such as publication and success in securing research grants) were seen to be strongly shaped by how priorities and opportunities are framed by funders and international experts. One participant, hinting at such wider drivers, reflected on the challenge of “*educating the donor ‘experts’ about what research is fascinating and important rather than pedestrian and ‘rational’*”.

### Establishing and retaining research teams

The clusters positioned to the lower right of Figure 1 developed analysis of challenges in the academic environment to support HRCS goals. This cluster showed clear linkage to the adjacent cluster ‘Addressing disincentives for academic engagement’ previously considered*.* Many comments focused on the notion of a ‘critical mass’ of researchers to establish viable capacity, and the barriers to achieving this. One participant noted “*the lack of career rewards for catalytic, synthetic, cross-disciplinary building versus private research productivity*”, with others reinforcing the notion with talk of “*piece-meal*”, “*project-based*”, and “*fragmented*” support as all barriers to effective team-building. Retention *per se* was noted as a challenge, but the major focus here was on coordinating efforts effectively with a goal – as one participant noted – of “*building research teams of young investigators as opposed to individual stars*”.

### Sustaining mentorship and institutional support

This cluster elaborated on the institutional requirements for developing research capacity. Issues of securing IT and laboratory facilities were noted, as were requirements for effective systems of research administration. However, the strongest emphasis was on issues of mentorship. ‘*Mentoring for novice researchers*’, the difficulty of finding time away from projects for ‘*intensive one-to-one mentoring*’ required, ‘*difficulty of supporting an apprenticeship process over time*’, ‘*finding appropriate mentors… there are simply too few*’, etc., represented the densest clustering of issues for the whole exercise.

## Discussion

Reflecting other recent analyses (e.g., [4, 28–31]), the above suggests something of the complexity of processes of health research capacity development, the many issues with which it engages, and the multiple pathways by which it may be supported. Landau, for example, notes the influence of “*fundamentally unequal resource endowments and incentive structures*” [28], p. 555 in undermining many well-intentioned initiatives, echoing two of the themes highlighted above. He also reflects on the challenges in Southern researchers being encouraged to focus principally on policy-oriented research given the role of Northern players in shaping such policy agendas, and thus the difficulty of Southern voices retaining ultimate authority over funding and research priorities. We join with Landau in suggesting that technocratic strategies to address health research capacity development issues have typically ignored the realities of “*the political economy of knowledge production*” [28], p. 558 that shapes such efforts.

In particular, reflecting upon the challenges and the documented experience of HRCS through the HGL work, we believe that there is a somewhat unique political economy influencing the shape of such efforts which has received inadequate recognition to date. Specifically, compared to other sectors and industries where capacity development is a pertinent issue, health research capacity development appears to be marked by three drivers outlined below.

### Independent researchers

The goal of capacitating ‘independent researchers’ is prominent within the discourse of health research capacity development. Despite some moves towards ‘team science’, the model of individual senior researchers leading research groups remains the dominant model of health research (and, crucially, major health funding mechanisms) in the northern hemisphere. Such ‘principal investigators’ are generally assumed to operate within a framework of academic freedom that provides them with the discretion and autonomy of individual research scientists. Transferred to a southern context, this model potentially fetishizes the development of ‘research leaders’, who then serve as (frequently over-committed) gatekeepers of resources and potential patronage. There are benefits of this model, but there are major challenges with it in relation to programmatically and policy oriented research. As one of the research leaders interviewed as part of the HGL program of work noted:

“*There’s a lot of goodwill from northern institutions, but the approach remains ‘in our own image’. I don’t think we hear enough Southern voices challenging this. African scientists, like others, have been socialized into this approach, and those that fit in this framework can be quite successful. But there needs to be a community of people who conceptualize research in a new manner. The ‘lone researcher’ model doesn’t work well in the usual work environment, where teams are much more likely to be successful. Systems still reinforce this notion through talk of ‘principal investigators’ and their demonstrating capacity for ‘independent research’. It’s amazing with all the resources that have been invested in building research capacity globally that there’s not a new way – and new voices – more clearly emerging*” (Wafaa El Sadr, [20]).

### Globalization of knowledge

It is increasingly recognized that the researcher autonomy noted above is exercised within a globalized ‘knowledge industry’. This industry is characterized by a free flow of knowledge products, remarkable mobility of researchers, and a global ‘community of science’ networking individuals. This development is potentially transformative of approaches to research capacity development, yet, to date, the focus has largely been on facilitation of knowledge sharing (through open access journals, for example) rather than implications for knowledge generation. Research implementation remains dominated by notions of geography, including in terms of ‘field sites’, for which local and international researchers have proprietorial (or semi-proprietorial) rights. Such rootedness in context has clear advantages for more culturally, epidemiologically, and clinically informed research, but the forces of globalization increasingly make such ‘local’ knowledge widely available and offer prospects for engagement in research freed from spatial affiliation.

Two other aspects of globalization significantly shape the political economy of health research. First, the development of metrics, such as those associated with the WHO Global Burden of Disease Study (and the common framing of the Demographic and Health Survey and Multi-Indicator Cluster Survey), clearly drive an agenda of cross-national comparison and global learning. Second, health research funding from multilateral and Northern funders is increasingly articulated with respect to the ‘global good’ of knowledge products, not just their local utilization (which is seen as the province of national governments and domestic funding sources).

### Institutionalization of capacity

Between the domain of the ‘sole trader’ independent researcher and the global market of knowledge production and transfer lies the domain of the institution. The institutionalization of capacity – within research institutes and universities – remains a key policy concern, both as a means to shape knowledge creation and to ensure (more) equitable or sustainable models of knowledge transfer. However, the above analysis suggests the complexity of such institutionalization. Within a globalized system, the ties of individual academics to institutions may be considered ‘tactical’ in terms of more or less durable mutual interests rather than *de facto* alliances determined by geographical requirements and labor market constraints. Institutions provide a ‘safe harbor’ for independent researchers to administer their research activities, and provide the broader academic environment (including students and teaching facilities) to enable their work. Researchers provide institutions the opportunity for profile and influence, both of which may support resource mobilization.

However, as noted above, the differential incentives operating for researchers and their institutions can make such relationships fractious. Case studies provided much evidence of trust between researchers (i.e., independent scientists within a globalized network) being perceived as much stronger than that between researchers and their institutions, or between institutions – where ‘institutions’ were seen as the overall body, usually a university, but sometimes a department or faculty. As we discuss below, the idea of ‘institution’ and, perhaps, the idea of a university, needs to be problematized within the global health research and global health literatures. Scholarship on higher education in the social sciences has for some time addressed the university as a social institution, the institutional realities of research and the changing roles of universities [32–34], but these perspectives have not been integrated into the conceptual and funding frameworks supporting global health research or research capacity strengthening. The emphasis on relationships, mentorship, and collegiality among the symposium participants and in several of the project case studies suggests that researchers do not see themselves primarily as ‘sole traders’. The values driving many of them – including teaching, building capacity in Africa, multidisciplinarity, policy engagement, and knowledge translation – also include loyalty to colleagues and need more than a convenient individual office or primarily electronic relationships to be realized: researchers with whom we interacted also value local institutional affiliations for regular face-to-face interactions with trusted colleagues.

### Implications of these three drivers of HRCS political economy

Strategies for HRCS that fail to acknowledge the complex agendas deriving from these three drivers are clearly likely to be ineffective. We suggest that many of the challenges noted in the above analysis stem from the competing influences of these drivers. For example, HRCS strategies couched in terms of institutional development are clearly at risk for ‘capture’ by independent researcher interests that are incentivized more strongly than institutional goals. Conversely, institutional strategies for capacity development that fail to acknowledge the ‘social capital’ of trust and collegiality between independent researchers linked through global or local, ‘infra-institutional’ interaction that enables and sustains partnership, makes them vulnerable to individual mobility (understood as the ‘South-North’ capacity strengthening noted above).

More generally, institutional agendas (and their potential conflict with the reciprocal values of a ‘community of science’ linking individual researchers in a shared research or public health agenda) appear to be infrequently appropriately problematized, perhaps particularly so in recent efforts to strengthen institutional research management capacity. These efforts represent a welcome recognition that there is a large gap between being a technically proficient and creative scientist on the one hand and being able to manage the large and complex teams, budgets, and partnerships increasingly characterizing health research on the other. However, they fail to recognize the equally large gap between corporate and institutional interests – and the attendant bureaucracies needed to administer ‘big science’ and large institutions – as well as the individual and team interests and often messy rhythms of research. Most universities are not managed like creative industries or biotech startups, and neither are most funders able to tolerate ambiguity and risk, not least because the current financial crisis further entrenches the most conservative and narrow understandings of ‘accountability’ [35]. The ‘*enabling environment’* and ‘*research culture’* many of the Symposium participants prioritized speak to the need for trust, flexibility, and innovation understood in terms of change and some risk rather than as a short-form for ‘potentially commercializable intellectual property’. Guidelines for research costing developed by a consortium of global health research funders [36] remind universities and research institutions that their research management and support systems should serve and support research and researchers, rather than the other way around. The HGL experiences shared above argue that this is not often the case.

Indeed, it could be argued that, at the institutional level, many African universities are likely to be at the worst point with respect to a flexible, responsive enabling environment: completely weak universities at least leave the researchers alone to get what funding they can and do what they want (at the risk of *per diem* and consultancy driven ‘survival research’); elite universities support and celebrate both local and globalized lone rangers, but mid-level or emerging universities, in trying to put systems in place and be ‘accountable’ to national agendas and foreign funders, run the risk of stifling initiative and productivity through managerialism and bureaucratization, which emphasize compliance over creativity and collegiality. In the process, they risk losing their most creative and productive researchers to the global knowledge economy because, in fact, the ‘individual researcher’ model is still the fundamental model of research.

This links also with national institutionalization efforts through attempts to negotiate national research agendas and national research systems. In an era of global flows of knowledge and research – with donor investment shaped by the expectation of securing ‘global public goods’ and national governmental investments in such agendas frequently below agreed targets to make them functional [37, 38] – such efforts frequently appear inadequately articulated conceptually and politically.

## Conclusions

The findings of the HGL program and our reflection above on the three drivers in the political economy of global health research and capacity suggest a continuing tension among competing imperatives that are shared by both the Southern and Northern researchers and managers with whom we have engaged: ‘embracing globalization of knowledge and the global ‘Community of Science” versus ‘respecting and protecting national and institutional agendas’. Because the global health research landscape remains so unevenly resourced both within countries and, especially, between the global North and the global South, these imperatives are not only in a creative or dynamic tension, but also are often in direct competition with each other. What we have found promising in response to this tension is an increasing, and progressively nuanced, recognition and understanding of the existence, causes, and implications of the uneven global health research and institutional playing field. This is marked by an apparently growing willingness to explore coherent, pragmatic models that try to balance these competing imperatives [21, 29, 30, 39]. This approach to global HRCS acknowledges globalization and mobility of knowledge and its producers, but with respect to a complex and unequal landscape both within and between countries and institutions. Both local contexts and global fluidity, both entrenched power relations and opportunities for autonomy, and both subversion and transformation are acknowledged to be at play, sometimes within the same setting.

The work of capacity strengthening and innovation happens in the interstices and relationships as much as in the formal structures and metrics of research. We have seen a wide range of more and less successful initiatives in an equally wide range of settings. Our reflections lead us to agree with analyses emphasizing complexity and emergence in thinking about and working to enhance organizational capacity for social change [29, 40]. However, we are concerned that enthusiasm for the much greater ‘face validity’ and explanatory power of complex adaptive systems thinking as an alternative to linear and ‘engineering’ models of organizational development not efface attention to the realities of political economy. Not every future is feasible: power, organizational, disciplinary, and political cultures, resources, and history all shape and constrain possibility. Yet the resonance of themes, challenges, and pivotal transformations across contexts as different as Burkina Faso and North America, and across institutional settings from research networks to universities both within and between countries, suggests that we may have more in common than we realize.

The HGL program of work sought, in part, to build a community of practice sharing and learning across our respective experiences. We found that discussions about broad themes rapidly grew too abstract and stale to capture the richness of context or usefully address the myriad ways in which a few themes play out on the ground. The globalization that seems most promising to us is one which brings these local specificities into conversation, creating temporary but recurrent spaces in which to reflect, analyze, adapt others’ experience, and sometimes find enough common ground to join efforts.

## Endnote

For access to all reports, briefs, and other documentation related to the HGL initiative go to: http://www.hivaids-uwc.org.za/index.php/publications/4-hiv-a-aids-research-centre/hiv-a-aids-research-centre/140-soph-briefs.

## Abbreviations

HGL: HRCS Global Learning program
HRCS: Health research capacity strengthening
IDRC: International Development Research Centre
MDS: Multi-dimensional scaling
UWC: University of Western Cape.
